# Characterization of a Novel Alginate Lyase with Two Alginate Lyase Domains from the Marine Bacterium *Vibrio* sp. C42

**DOI:** 10.3390/md20120746

**Published:** 2022-11-26

**Authors:** Xiao-Meng Sun, Zhao Xue, Mei-Ling Sun, Yi Zhang, Yu-Zhong Zhang, Hui-Hui Fu, Yu-Qiang Zhang, Peng Wang

**Affiliations:** 1 College of Marine Life Sciences, Frontiers Science Center for Deep Ocean Multispheres and Earth System, Ocean University of China, Qingdao 266003, China; 2 State Key Laboratory of Microbial Technology, Marine Biotechnology Center, Shandong University, Qingdao 266237, China; 3 Life Science College, Shandong Normal University, Jinan 250014, China

**Keywords:** alginate lyase, catalytic domain, alginate degradation, PL7 lyase, *Vibrio*, marine bacterium

## Abstract

Alginate is abundant in the cell walls of brown algae. Alginate lyases can degrade alginate, and thus play an important role in the marine carbon cycle and industrial production. Currently, most reported alginate lyases contain only one functional alginate lyase domain. AlyC8 is a putative alginate lyase with two alginate lyase domains (CD1 and CD2) from the marine alginate-degrading strain *Vibrio* sp. C42. To characterize AlyC8 and its two catalytic domains, AlyC8 and its two catalytic domain-deleted mutants, AlyC8-CD1 and AlyC8-CD2, were expressed in *Escherichia coli*. All three proteins have noticeable activity toward sodium alginate and exhibit optimal activities at pH 8.0–9.0 and at 30–40 °C, demonstrating that both CD1 and CD2 are functional. However, CD1 and CD2 showed opposite substrate specificity. The differences in substrate specificity and degradation products of alginate between the mutants and AlyC8 demonstrate that CD1 and CD2 can act synergistically to enable AlyC8 to degrade various alginate substrates into smaller oligomeric products. Moreover, kinetic analysis indicated that AlyC8-CD1 plays a major role in the degradation of alginate by AlyC8. These results demonstrate that AlyC8 is a novel alginate lyase with two functional catalytic domains that are synergistic in alginate degradation, which is helpful for a better understanding of alginate lyases and alginate degradation.

## 1. Introduction

Brown algae are one of the important sources of marine primary productivity and play a momentous role in the carbon cycle of the marine ecosystem [1]. Alginate is an acidic polysaccharide that widely exists in the cell walls of brown algae, accounting for 30–60% of the dry weight of brown algae [2]. Alginate consists of β-D-mannuronate (M) and its C5 epimer, α-L-guluronate (G), linked homogeneously or heterogeneously by 1→4 glycosidic bonds [3]. According to the sequence of monomers, alginate can be divided into three different blocks: polymanuronate (PM) blocks, polyguluronate (PG) blocks and polyMG/GM (PMG) blocks [4]. Alginate has been widely used as a stabilizer, emulsifier, thickener, and an additive in the food, cosmetic and pharmaceutical industries [3,5,6]. For example, alginate wound dressings can maintain a physiologically moist microenvironment, and facilitate wound healing [7]. Alginate hydrogels can protect probiotics [8], drug molecules [7] and unstable active compounds in cosmetics [9] against unfavorable conditions to increase the products’ efficacy.

Alginate lyase is the enzyme degrading alginate, operating via a β-elimination reaction to produce 4-deoxy-L-*erythro*-hex-4-enopyranosyluronic acid at the non-reducing end [10]. Alginate lyases have been isolated from a wide range of sources, including marine algae [11], marine mollusks [12], marine and terrestrial bacteria [13,14], fungi [15] and viruses [16]. According to the protein sequence, alginate lyases are distributed in the polysaccharide lyase (PL) families 5, −6, −7, −8, −14, −15, −17, −18, −31, −32, −34, −36, −39 and −41 in the Carbohydrate-Active Enzymes (CAZy) database (http://www.cazy.org/, accessed on 10 October 2022) [17,18]. Differences in protein sequence and structure allow alginate lyases to have different substrate specificity. They can be divided into three types depending on the substrate specificity. The first type can specifically degrade PG (EC 4.2.2.11), the second type can specifically degrade PM (EC 4.2.2.3) and the third type can degrade PG, PM and PMG (EC 4.2.2.-) [2]. Depending on the action mode, they can be classified into the endotype lyases producing oligosaccharides by cleaving the inside-chain glycosidic bonds [19] and the exotype lyases producing monomers or dimers by gradual degradation from the end of the alginate polymer [20,21]. Alginate lyases have wide application prospects in alginate structure research [22], protoplast preparation [23], AOs preparation [24], seaweed waste disposal [24], medical treatment [25] and biofuel preparation [26]. 

To date, more than 100 alginate lyases with diverse domain compositions have been reported. For instance, in addition to the C-terminal catalytic domain, the alginate lyase TsAly7B contains two N-terminal carbohydrate-binding modules (CBMs) that can enhance the thermostability of TsAly7B [27]. In addition to the N-terminal catalytic domain, alginate lyase AlyGC contains an extra C-terminal domain essential for AlyGC dimerization [28]. However, most of the multimodular alginate lyases reported so far contain just a single functional catalytic domain. Only one enzyme (A1-I) from *Sphingomonas* sp. strain A1, with two functional alginate lyase domains consisting of A1-II (PL7) and A1-III (PL5), has been reported [29,30,31].

In this study, a PL7 alginate lyase, AlyC8, with two putative catalytic domains, was identified from a marine alginate-degrading bacterium, *Vibrio* sp. C42, isolated from a brown algae *Sargassum* sample collected from the seashore of Shandong Province, China. Based on the sequence analysis, the two catalytic domains of AlyC8 (CD1 and CD2) both belong to the PL7 family but share no sequence similarity. AlyC8 and its catalytic domain-truncated mutants, AlyC8-CD1 and AlyC8-CD2, were then heterologously expressed and purified, and their alginolytic activities were confirmed. Moreover, their enzymatic properties were biochemically characterized, and the two domains showed synergy based on the biochemical analysis. The results provide a better understanding of alginate lyases and alginate degradation.

## 2. Results and Discussion

### 2.1. Sequence Analysis of AlyC8

**Figure 1:**
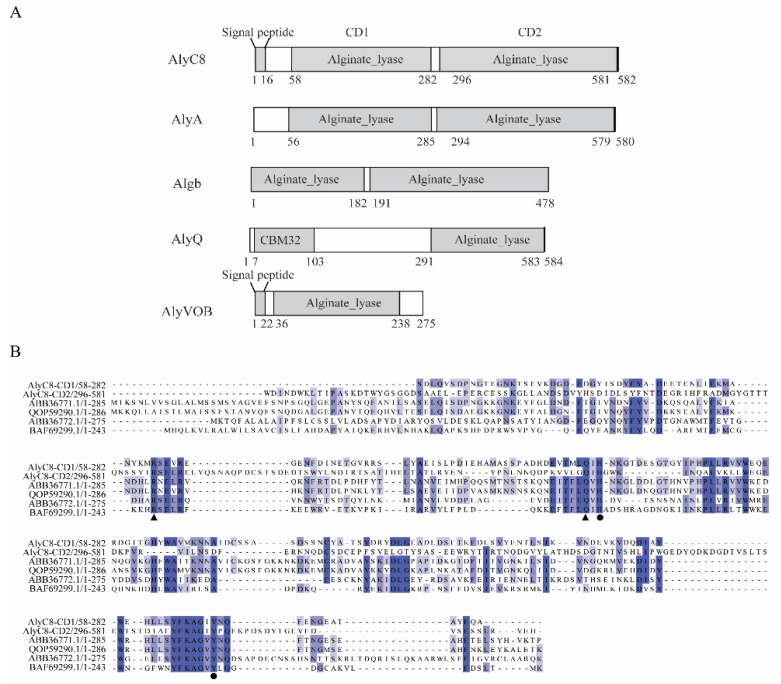

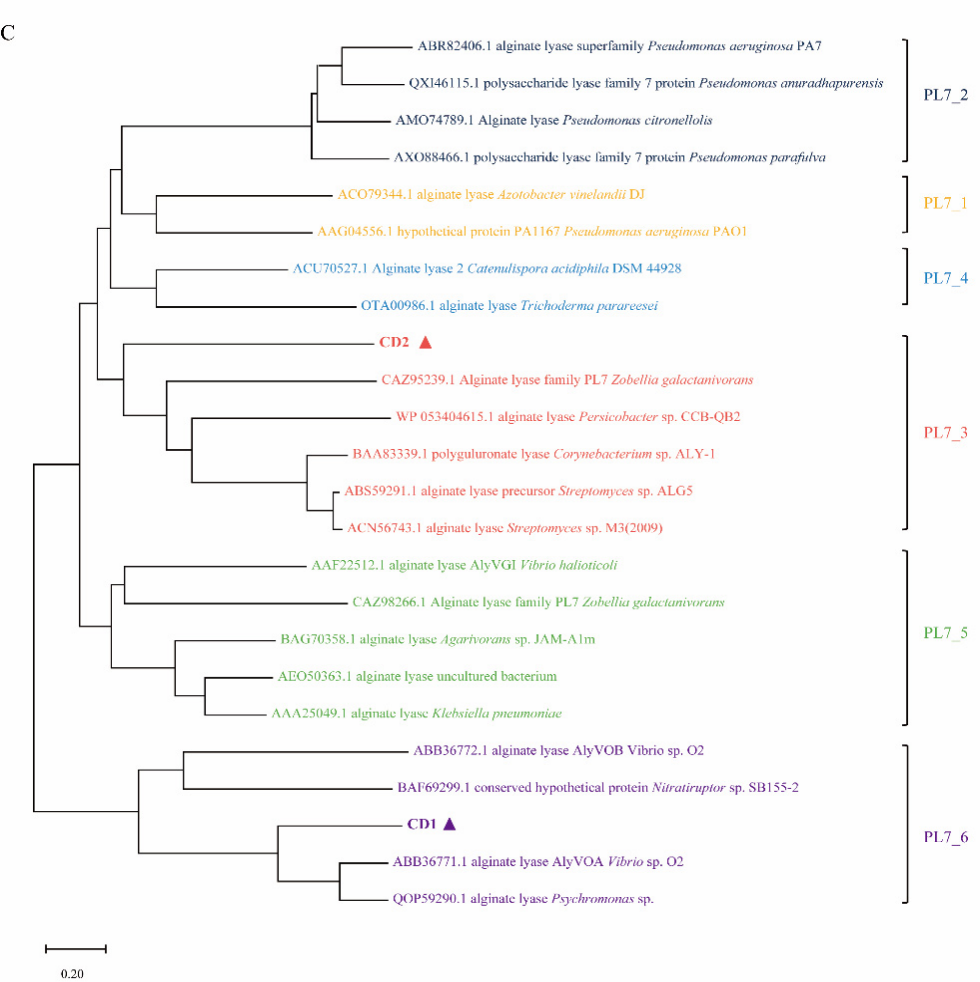
Sequence analysis of AlyC8. (**A**) Schematic domain diagram of the alginate lyase AlyC8 (GenBank: MBY7662542.1) and other PL7 alginate lyases. AlyA (GenBank: EAP94921.1) is from *Vibrio splendidus* 12B01, Algb (GenBank: KM507331) from *Vibrio* sp. W13, AlyQ (GenBank: WP_053404615.1) from *Persicobacter* sp. CCB-QB2 and AlyVOB (GenBank: ABB36772.1) from *Vibrio* sp. O2. The signal peptide was predicted by SignalP 5.0 Server. The conserved domains were analyzed by Conserved Domain Database. CBM32, the carbohydrate-binding module family 32 domain. (**B**) Multiple sequence alignment of the CD1 and CD2 domains with other characterized PL7 alginate lyases. The amino acid residues involved in catalysis and those neutralizing the negative charge are marked with spots and triangles, respectively. (**C**) Phylogenetic analysis of CD1 and CD2 with other alginate lyases from the PL7 family by using the neighbor joining method. Bootstrap analysis of 1000 replicates was conducted.

Based on gene annotation of the genome, strain *V.* sp. C42 contains 11 putative alginate lyases (GenBank: JAEKGD000000000.1). The gene *alyC8* is 1749 bp in length and encodes a putative alginate lyase AlyC8 of 582 amino acid residues with a predicted 16-residue signal peptide (Figure 1A). According to the blast result against the NCBI conserved domain database, AlyC8 contains two alginate lyase domains: the CD1 domain (Ser58-Ala282) and the CD2 domain (Trp296-His581), both belonging to the PL7 family. Nevertheless, though the amino acid residues that may be involved in catalysis of the CD1 and CD2 domains are identical based on the sequence alignment with other characterized PL7 alginate lyases (Figure 1B), the CD1 and CD2 domains share quite low sequence similarity (22.4% identity under 89.3% sequence coverage). Phylogenetic analysis showed that CD1 and CD2 belong to subfamily 6 and subfamily 3 of the PL7 family (Figure 1C), respectively, implying that they may have different properties. The closest sequence homologue to AlyC8 is AlyA (90.38% identity), which also possesses two separate putative alginate lyase modules, albeit one of them is not functional [32].

### 2.2. Activity Determination of AlyC8, AlyC8-CD1 and AlyC8-CD2

**Figure 2:**
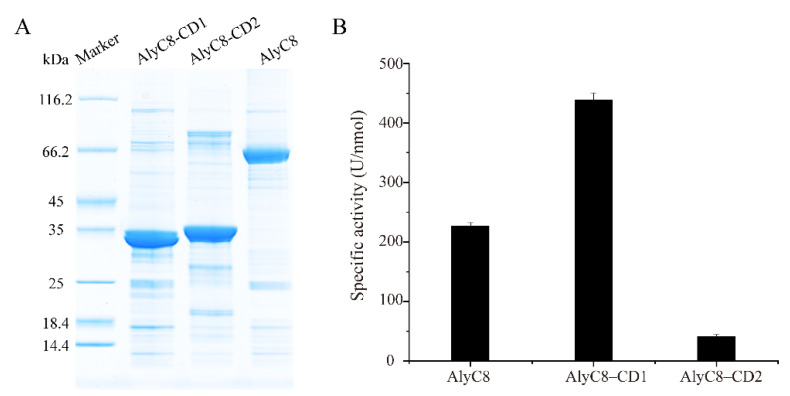
Determination of the activity of AlyC8, AlyC8-CD1 and AlyC8-CD2. (**A**) SDS-PAGE analysis of the purified AlyC8 and its mutants AlyC8-CD1 and AlyC8-CD2. (**B**) Alginate lyase activities of AlyC8, AlyC8-CD1 and AlyC8-CD2. The reactions were carried out at 30 °C for 10 min in a 200 µL mixture containing 20 µL enzyme, 2.0 mg/mL sodium alginate, 0.5 M NaCl and 50 mM Tris-HCl (pH 8.0). The graphs show data from triplicate experiments (mean ± standard deviation [SD]).

To characterize AlyC8, we heterologously expressed it in *Escherichia coli* BL21 (DE3) and purified the recombinant AlyC8 protein. SDS-PAGE analysis showed that the recombinant AlyC8 was successfully purified and its molecular mass was approximately 65 kDa, corresponding to its theoretical molecular mass (65.4 kDa) (Figure 2A). The alginate lyase activity of AlyC8 was verified with an enzymatic activity assay (Figure 2B), demonstrating that AlyC8 is an alginate lyase.

Afterwards, to determine whether both domains of AlyC8 were alginolytic, we constructed two truncated mutants, AlyC8-CD1 (Ser58-Ile290), lacking the CD2 domain, and AlyC8-CD2 (Ile290-His581), lacking the CD1 domain, and measured their alginate lyase activity. The purified AlyC8-CD1 and AlyC8-CD2 had approximate molecular masses of 32 kDa and 34 kDa, respectively, corresponding to their theoretical molecular masses (31.7 kDa for AlyC8-CD1 and 32.7 kDa for AlyC8-CD2) (Figure 2A). The activity assay showed that both AlyC8-CD1 and AlyC8-CD2 could degrade alginate (Figure 2B), demonstrating that AlyC8 contains two functional alginate lyase domains. 

At present, three alginate lyases have been reported to contain two alginate lyase domains in the CAZy database, namely Algb from *Vibrio* sp. W13 [13], AlyA from *Vibrio splendidus* 12B01 [32] and A1-I from *Sphingomonas* sp. strain A1 [31]. However, for AlyA, only its C-terminal alginate lyase domain shows the alginate lyase activity, while its N-terminal domain has no activity [32]. As for Algb, only the alginate lyase activity of the full-length enzyme was measured; as such, the activities of its two putative catalytic domains have not been independently investigated [13]. A1-I is a precursor protein consisting of a PL5 domain (A1-III) and a PL7 domain (A1-II). A1-I was found to undergo a self-processing reaction, being converted into A1-II and A1-III [31], but such a phenomenon was not observed on AlyC8. As shown in Figure 2A, the purified AlyC8 contains both the CD1 and CD2 domains based on its molecular mass. Thus, AlyC8 is a novel alginate lyase with two catalytic domains, both capable of decomposing alginate.

### 2.3. Effects of Temperature and pH on the Activities of AlyC8, AlyC8-CD1 and AlyC8-CD2

**Figure 3:**
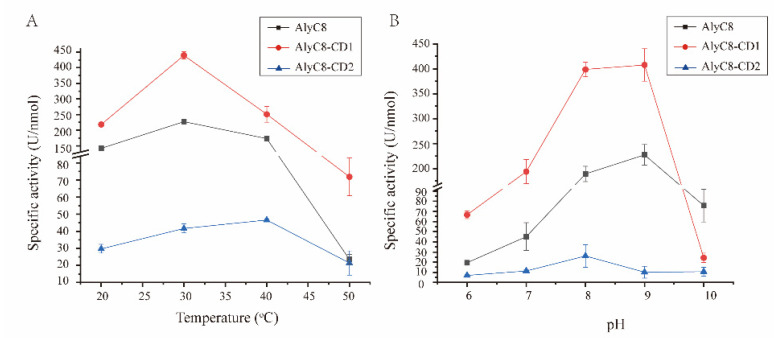
Effects of temperature (**A**) and pH (**B**) on the activities of AlyC8, AlyC8-CD1 and AlyC8-CD2. Experiments in (**A**) were conducted for 10 min in a 200-µL mixture containing 20 µL enzyme, 2.0 mg/mL sodium alginate, 50 mM Tris-HCl and 0.5 M NaCl at 20–50 °C under the optimum pH of each enzyme. Experiments in (**B**) were conducted for 10 min in a 200 µL mixture containing 20 µL enzyme, 2.0 mg/mL sodium alginate, 50 mM Britton–Robinson (pH 6.0–10.0) and 0.5 M NaCl at the optimum temperature of each enzyme. The graphs show data from triplicate experiments (mean ± standard deviation [SD]).

To investigate the functions of the CD1 and CD2 domains in the full-length enzyme, we investigated the biochemical properties of AlyC8, AlyC8-CD1 and AlyC8-CD2. Firstly, we measured the effects of temperature and pH on their alginate lyase activities. The optimal enzymatic reaction temperature of AlyC8 and AlyC8-CD1 was the same, at 30 °C, while it was 40 °C for AlyC8-CD2. All three enzymes retained more than 50% of the maximum activity in the range from 20 °C to 40 °C, and their activity decreased markedly when the temperature reached 50 °C (Figure 3A). At 40 °C, AlyC8 showed higher relative activity (76.03% of its highest enzyme activity) compared with AlyC8-CD1 (57.15% of its highest enzyme activity), probably due to the high activity of AlyC8-CD2 at 40 °C. As shown in Figure 3B, both AlyC8 and AlyC8-CD1 showed their highest activities at pH 9.0, whereas for AlyC8-CD2 it was at pH 8.0. AlyC8, AlyC8-CD1 and AlyC8-CD2 all retained more than 40% of the highest activity at the pH range from 8.0 to 9.0 (Figure 3B).

The enzyme activities of alginate lyases are usually related to the living environment of their sources. For example, the PL7 alginate lyases AlyA, AlyB, AlyD and AlyE from the marine bacterium *Vibrio splendidus* 12B01 showed their highest activities at 20–25 °C and pH 7.0–8.0. They retained more than 50% of their maximum activity in the range of 10–25 °C and pH 7.0–8.5 [32]. The PL6 alginate lyase ALFA4 from the marine bacterium *Formosa algae* KMM 3553 had the highest activity at 30 °C and pH 8.0. It maintained approximately 50% of the maximum activity in the range of 25–40 °C and lost more than 60% of the maximum activity under other conditions [33]. The PL5 alginate lyase algA from the marine bacterium *Pseudomonas* sp. E03 had a maximum activity at pH 8.0 and 30 °C and maintained more than 60% of the maximum activity between pH 7.0 and 9.0 and 20–40 °C [34].

In general, most of the marine-derived alginate lyases from different families showed higher enzyme activity (more than 50% of the maximum activity) between 20 and 40 °C and at pH 7.0–9.0, reflecting their adaptation to the marine environment. Similarly, AlyC8, AlyC8-CD1 and AlyC8-CD2 showed high catalytic activities between 20 and 40 °C and at pH 8.0–9.0, indicating that they can adapt well to the low temperature and alkalescent marine environment.

### 2.4. Thermal Stability of AlyC8, AlyC8-CD1 and AlyC8-CD2

**Figure 4:**
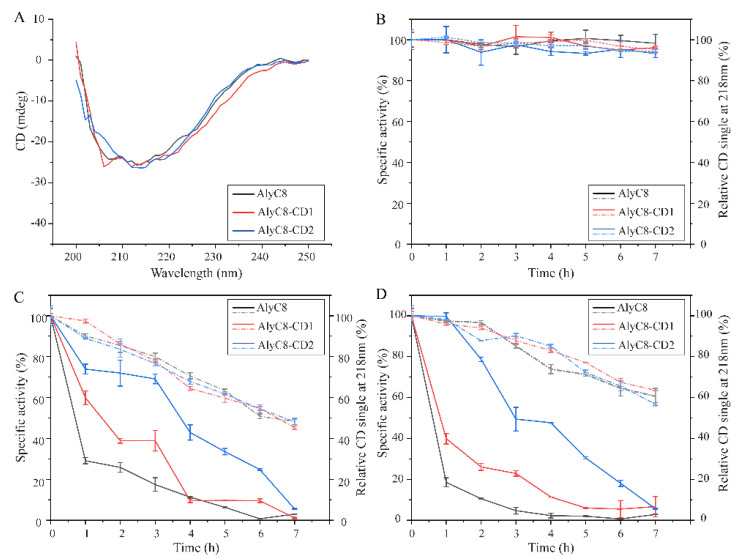
Thermal stability of AlyC8, AlyC8-CD1 and AlyC8-CD2. (**A**) CD spectra of AlyC8 and its mutants AlyC8-CD1 and AlyC8-CD2. (**B**) Relative specific activity and relative CD signal at 218 nm of AlyC8, AlyC8-CD1 and AlyC8-CD2 incubated at 0 °C. (**C**) Relative specific activity and relative CD signal at 218 nm of AlyC8, AlyC8-CD1 and AlyC8-CD2 incubated at 30 °C. (**D**) Relative specific activity and relative CD signal at 218 nm of AlyC8, AlyC8-CD1 and AlyC8-CD2 incubated at 40 °C. In (**B**), (**C**) and (**D**), the relative specific activity and relative CD signal at 218 nm are shown as continuous lines and dashed lines, respectively. The reactions in (**B**), (**C**) and (**D**) were conducted for 10 min in a 200 µL mixture containing 20 µL enzyme, 2.0 mg/mL sodium alginate, 50 mM Tris-HCl and 0.5 M NaCl at the optimum reaction conditions of each enzyme. The graphs show data from triplicate experiments (mean ± standard deviation [SD]).

Since AlyC8 consists of CD1 and CD2 connected by a linker, we investigated the effect of temperature over time on the thermal stability of AlyC8, AlyC8-CD1 and AlyC8-CD2, by analyzing the residual alginate lyase activity and the residual secondary structures of these enzymes. PL7 alginate lyase structures are mainly composed of β-sheets [35,36]. The negative extremum of the circular dichroism (CD) spectra of AlyC8, AlyC8-CD1 and AlyC8-CD2 was at 218 nm (Figure 4A), consistent with that of the reported PL7 lyases [37]. As shown in Figure 4B, both the structures and activities of AlyC8, AlyC8-CD1 and AlyC8-CD2 were stable for at least 7 h at 0 °C. When exposed to 30 °C or 40 °C, all three enzymes showed similar changes in the secondary structure from 0 to 7 h, but different changes in the residual alginate lyase activity. In general, when incubated at the same temperature for the same time period, AlyC8-CD2 retained the highest residual activity, followed by AlyC8-CD1, then AlyC8, which retained the lowest (Figure 4C,D). These results demonstrated that, for activity, AlyC8-CD2 has the best thermal stability, followed by AlyC8-CD1, and then AlyC8, which is the most thermolabile. Considering that the two catalytic domains of AlyC8 are connected by a linker, we hypothesize that the linker is flexible and undergoes rapid changes when the temperature increases, leading to AlyC8 instability. Noticeably, after being exposed to 30 °C for 7 h, all three enzymes retained more than 50% of their secondary structure, but lost almost all of their alginate lyase activities (Figure 4C), which suggested that the loss of their alginate lyase activities was not entirely caused by the disruption of their secondary structures.

### 2.5. Substrate Specificities of AlyC8, AlyC8-CD1 and AlyC8-CD2

**Figure 5:**
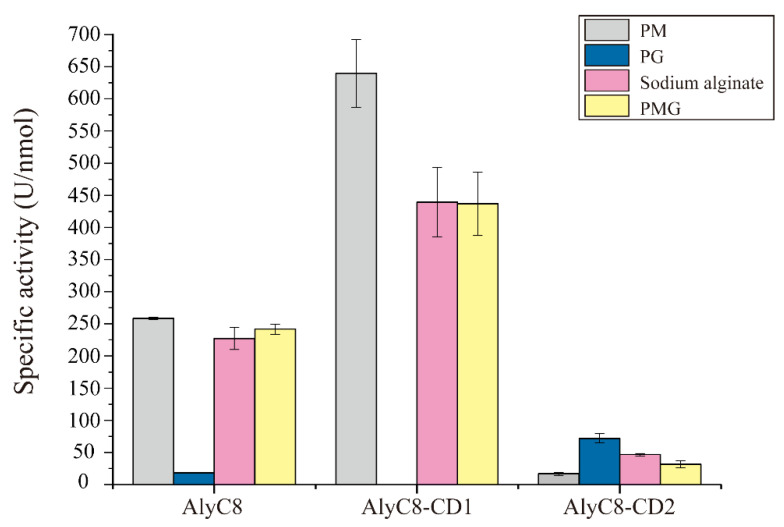
The substrate specificities of AlyC8, AlyC8-CD1 and AlyC8-CD2. Substrate specificities of the enzymes were measured toward polymannuronate (PM), polyguluronate (PG), sodium alginate and PMG. Experiments were conducted for 10 min in a 200 µL mixture containing 20 µL enzyme, 2.0 mg/mL substrate, 50 mM Tris-HCl and 0.5 M NaCl at the optimum reaction conditions of each enzyme. The graph shows data from triplicate experiments (mean ± standard deviation [SD]).

It has been reported that alginate lyases of different subfamilies may have different substrate specificities [36]. Therefore, we analyzed the substrate specificities of AlyC8-CD1 and AlyC8-CD2, which belong to different PL7 subfamilies (Figure 1B), and also AlyC8, towards four different alginate substrates (PM, PG, PMG and sodium alginate) (Figure 5). AlyC8-CD1 showed high activities towards PM, PMG and sodium alginate, with a preference for PM, which is consistent with the substrate specificity of the subfamily 6 lyases [36]. However, it had no activity towards PG. Contrastingly, AlyC8-CD2 had the highest activity towards PG and the lowest activity towards PM, consistent with the substrate specificity of the subfamily 3 lyases [38,39]. Furthermore, AlyC8 was active towards all alginate substrates, and notably, it had detectable activity towards PG, which should benefit from the significant activity of AlyC8-CD2 towards PG. Therefore, the complementary substrate specificities of AlyC8-CD1 and AlyC8-CD2 enable the full-length enzyme to degrade all types of glycosidic bonds occurring in alginate.

### 2.6. Kinetic Analysis of AlyC8-CD1 and AlyC8-CD2

The kinetic parameters of AlyC8-CD1 and AlyC8-CD2 at their optimal conditions were analyzed (Table 1). The *K*_m_ value of AlyC8-CD1 was lower than that of AlyC8-CD2, indicating that the affinity for sodium alginate of AlyC8-CD1 was higher than that of AlyC8-CD2. The *V*_max_ value of AlyC8-CD1 was much higher than that of AlyC8-CD2, demonstrating that the catalytic rate of AlyC8-CD1 was higher than that of AlyC8-CD2. In addition, the *k*_cat_*/K*_m_ value of AlyC8-CD1 was also higher than that for AlyC8-CD2, indicating that AlyC8-CD1 had a higher catalytic efficiency. In conclusion, compared to AlyC8-CD2, AlyC8-CD1 has a higher affinity, catalytic rate and catalytic efficiency for sodium alginate, suggesting that the CD1 domain plays a major role in alginate degradation by the full-length enzyme.

### 2.7. The Synergy between AlyC8-CD1 and AlyC8-CD2

**Figure 6:**
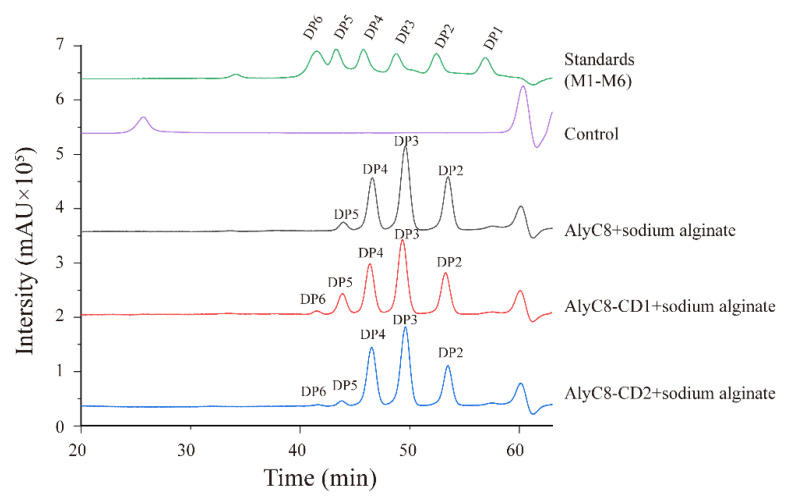
The final degradation product analysis of AlyC8, AlyC8-CD1 and AlyC8-CD2. Experiments were conducted for 24 h in a 200 µL mixture containing 1 nmol/mL enzyme, 2 mg/mL sodium alginate, 50 mM Tris-HCl and 500 mM NaCl at the optimum pH and temperature of each enzyme. The products were analyzed by high performance liquid chromatography using a Superdex peptide 10/300 GL column monitored at a wavelength of 210 nm. The control was treated with pre-heated inactivated lyases. Saturated mannuronate monomer and oligosaccharides at DP2 to DP6 were taken as the standards. DP, degree of polymerization.

We further investigated the final degradation products of AlyC8, AlyC8-CD1 and AlyC8-CD2 activity on sodium alginate (Figure 6). AlyC8, AlyC8-CD1 and AlyC8-CD2 all degraded alginate by an endolytic mode, producing a variety of oligosaccharides as the products, among which trisaccharides were the most predominant. For AlyC8-CD1, the final products contained oligosaccharides with degrees of polymerization (DPs) ranging from 2 to 6. The products of AlyC8-CD2 were similar to those of AlyC8-CD1, also containing oligosaccharides ranging from DP2 to DP6. Nevertheless, the degradation products of AlyC8 contained only oligosaccharides ranging from DP2 to DP5. These results implied that the combined activities of CD1 and CD2 led to the degradation of alginate into smaller saccharides. The sizes of the degradation products of AlyC8-CD1 and AlyC8-CD2 are mainly related to the amino acid residues involved in the substrate recognition and binding at their binding sites [40,41]. Therefore, though AlyC8-CD2 has activities towards different substrates, it cannot further degrade some hexamers into smaller ones. As aforementioned, the CD1 and CD2 domains share low sequence identity and exhibit complementary substrate selectivity, indicating that the residues involved in substrate recognition and binding in their catalytic centers may be different. Due to these differences, the hexamers that cannot be degraded by the CD2 domain may be further degraded by the CD1 domain and vice versa, leading to the disappearance of hexamers from the final products of AlyC8. Nevertheless, the molecular mechanism for the synergy of the CD1 and CD2 domains still needs further investigation.

Many studies have revealed that an alginate-degrading strain usually secretes multiple alginate lyases with different biochemical characteristics to synergistically degrade alginate [15,42]. The CD1 and CD2 domains of AlyC8 showed a synergistic effect, reflecting the broader alginate substrate selectivity and smaller oligomer products, which is a novel kind of synergism different from the reported ones between multiple alginate lyases.

Currently, some carbohydrate-degrading enzymes consisting of multiple catalytic domains have been reported, and several reports have suggested that these enzymes may be more powerful in degrading polysaccharides due to the possible intermolecular and intramolecular synergism between their catalytic domains. For instance, CmChi3, which contains two glycoside hydrolase family 18 (GH18) catalytic domains with different enzymatic activities, can completely degrade colloidal chitin to yield N-acetyl-D-glucosamine as the sole end product by its endochitinase, N-acetyl-β-d-glucosaminidase and transglycosylase activities [43]. In addition a multimodular glycoside hydrolase which contains a GH10 and a GH48 domain can hydrolyze cellulose and xylan by intramolecular and intermolecular synergy, respectively [44]. Finally, CelYZ, an artificial enzyme generated by fusing the GH48 and CBM domains of CelY with the GH9 and CBM domains of CelZ into a single polypeptide, shows an improvement of cellulase activity compared to both the single enzymes [45]. Therefore, the two catalytic domains in a single enzyme may affect each other, and the interaction may have an impact on their overall functions [44]. Alternatively, the proximity of two synergistic catalytic domains in a single enzyme may further amplify the synergistic effect, enabling multimodular enzymes with enhanced abilities to degrade the substrates.

## 3. Conclusions

Alginate lyases play important roles in the marine carbon cycle, as well as in biotechnology and industry. However, until now, only one study of an alginate lyase with two functional catalytic domains has been reported. In this study, we characterized a novel PL7 alginate lyase, AlyC8, with two functional catalytic domains from an alginate-degrading strain *V.* sp. C42 isolated from a *Sargassum* sample. The two alginate lyase catalytic domains of AlyC8, CD1 and CD2, belong to different PL7 subfamilies. CD1 and CD2 show a significant difference in substrate selectivity and have synergistic effects in alginate degradation, probably due to the complementarity of substrate specificity. This study provides a better understanding of alginate lyases and alginate degradation. Meanwhile, the synergy between the two catalytic domains of AlyC8 gives clues to the construction of a single-enzyme catalytic system that may replace the enzyme cocktail system in industrial production, which can promote the degradation of alginate in a more efficient, synergistic manner, reduce the cost and simplify the conversion process.

## 4. Materials and Methods

### 4.1. Materials and Strains

The alginate-degrading strain *V.* sp. C42 isolated from a *Sargassum* sample collected from coastal seawater in Shandong Province, China, was preserved in our lab. All the chemicals and reagents used in this research are of the highest level of purity available in China. Sodium alginate (purity: ≥98%) derived from brown algae was purchased from Sigma (Saint Louis, MO, USA). PM (6–8 kDa, purity: ≥98%), PG (6–8 kDa, purity: ≥98%) and alginate oligosaccharides (purity: ≥97%) were acquired from BZ Oligo Biotech Co., Ltd (Qingdao, China). PMG was prepared as previously described [46]. *Escherichia coli* strains DH5α and BL21 (DE3) were from Tsingke (Qingdao, China) and grown in Lysogeny broth (LB) medium with 100 µg/mL ampicillin at 37 °C.

### 4.2. Bioinformatics

The genomic DNA of *V.* sp. C42 was shotgun-sequenced on the Illumina Hiseq sequencing platform (Majorbio, Shanghai, China) and deposited in NCBI under the accession number of JAEKGD000000000.1. The putative alginate lyases in *V.* sp. C42 were predicted by dbCAN meta server (https://bcb.unl.edu/dbCAN2/blast.php, accessed on 10 October 2022) [47] and the residues encoding the putative signal peptide of the lyase were predicted by the SignalP-5.0 Server (https://services.healthtech.dtu.dk/service.php?SignalP-5.0, accessed on 10 October 2022) [48]. The theoretical molecular masses were analyzed on the ExPASy Server (https://web.expasy.org/protparam/, accessed on 10 October 2022 ) [49]. The conserved domains were analyzed by the Conserved Domain Database (CDD, https://www.ncbi.nlm.nih.gov/Structure/cdd/wrpsb.cgi, accessed on 10 October 2022) [50]. The phylogenetic tree was constructed based on the amino acid sequences by the neighbor-joining method using Mega X (Auckland, New Zealand) [51], and the bootstrap values of each branch of the phylogenetic tree were tested by 1000 repetitions.

### 4.3. Gene Cloning and Mutagenesis

Gene *alyC8* (GenBank: MBY7662542.1), without the signal peptide-encoding sequence, was amplified via PCR with the restriction sites *NdeI* and *XhoI* and cloned into the vector pET-22b that contained a C-terminal His-tag. The primers that were used are shown in Table 2. The cycling conditions for amplification are as follows: initial denaturation at 95 °C for 2 min, 30 cycles of 20 s denaturation at 95 °C, annealing at 52 °C for 20 s, and extension at 72 °C for 90 s. The final extension step was at 72 °C for 5 min. The truncated mutants, AlyC8-CD1 (Ser58-Ile290 of AlyC8) and AlyC8-CD2 (Ile290-His581 of AlyC8), were constructed using the same method.

### 4.4. Protein Expression and Purification

Recombinant AlyC8 and its mutants were expressed in *E. coli* BL21 (DE3), which was grown at 37 °C and 180 rpm in LB broth containing 100 µg/mL ampicillin. When the OD_600_ reached 0.6, induction was performed at 15 °C and 100 rpm under 0.3 mM isopropyl-β-D-thiogalactopyranoside (IPTG) for 16 h. After cultivation, the cells were harvested by centrifuged at 4 °C and 4000× *g* for 10 min and disrupted by a JN-02C French press (JNBIO, Guangzhou, China) in a buffer containing 50 mM Tris-HCl (pH 8.0) and 100 mM NaCl. Then, the resultant solution was centrifuged at 4 °C and 12,000× *g* for 60 min, and the supernatants were collected. The target proteins in the supernatants were purified by NTA-Ni Sepharose affinity chromatography (Qiagen, Germantown, Germany). The elution fraction was desalted using a disposable PD-10 column (GE Healthcare, Pittsburgh, PA, USA) with the buffer containing 10 mM Tris-HCl (pH 8.0) and 100 mM NaCl. The purified samples were analyzed by SDS-PAGE. The concentrations of proteins were quantified by the bicinchoninic acid (BCA) protein assay kit (Thermo, Waltham, MA, USA).

### 4.5. Biochemical Characterization of AlyC8 and Its Mutants

The alginate lyase activity was measured by the ultraviolet absorption spectrometry method [3]. Briefly, the enzymatic activity assay was performed in a 200 µL reaction system which contained enzyme (AlyC8, 5.56 µg/mL; AlyC8-CD1, 9.34 µg/mL; AlyC8-CD2, 6.08 µg/mL), 50 mM Tris-HCl, 500 mM NaCl and 2 mg/mL substrate. The mixture was incubated at the optimum temperature and pH of each enzyme for 10 min. The reaction was terminated by boiling the mixture for 10 min, and the alginate lyase activity was measured by monitoring the absorbance of the reaction solution at 235 nm (A_235_), which was caused by the production of unsaturated uronic acids in the mixture. One unit (U) of enzyme activity was defined as the amount of enzyme required to produce an increase of 0.1 per minute at 235 nm. 

The optimum temperature for AlyC8, AlyC8-CD1 and AlyC8-CD2 activity was determined at their optimum pH (pH 9.0 for AlyC8, pH 9.0 for AlyC8-CD1 and pH 8.0 for AlyC8-CD2) in a range from 20 °C to 50 °C at intervals of 10 °C in a buffer containing 50 mM Tris-HCl and 500 mM NaCl. The optimum pH for AlyC8, AlyC8-CD1 and AlyC8-CD2 activity was determined at their optimum temperature (30 °C for AlyC8, 30 °C for AlyC8-CD1 and 40 °C for AlyC8-CD2) in the Britton–Robinson (B-R) buffer (40 mM boric acid, 40 mM acetic acid and 40 mM phosphoric acid, adjusted to different pH with 0.2 M NaOH) ranging from pH 6.0 to 10.0. The substrate specificity of AlyC8, AlyC8-CD1 and AlyC8-CD2 was determined at their optimum reaction conditions with PM, PG, PMG or sodium alginate as the substrate. The thermal stability of AlyC8 and its mutants were tested according to the method of Orlando et al. [52]. The residual alginate lyase activities of AlyC8 and its mutants were determined after the enzymes were incubated at 0, 30 or 40 °C for 1 h up to 7 h in the buffer containing 50 mM Tris-HCl (pH 8.0) and 500 mM NaCl.

### 4.6. Circular Dichroism Spectra

The overall secondary structures of AlyC8 and its mutants at a concentration of 0.5 mg/mL in the buffer containing 50 mM Tris-HCl (pH 8.0) and 500 mM NaCl were monitored at 25 °C on a J-810 CD spectropolarimeter (JASCO, Tokyo, Japan). CD spectra were collected from 200 to 250 nm at a scanning rate of 200 nm/min with a path length of 0.1 cm.

### 4.7. Degradation Products of the Alginate Lyases

The degradation products released from sodium alginate by the alginate lyases were analyzed by high performance liquid chromatography (HPLC) with purchased saturated mannuronic saccharides as the standards. The 200 µL reaction mixture containing 1 nmol/mL enzyme, 2 mg/mL substrate, 50 mM Tris-HCl and 500 mM NaCl was incubated for 24 h at the optimum pH and temperature for each enzyme. The reaction was terminated by adding 0.4 M trichloroacetic acid (TCA), and the degradation products were separated on a Superdex Peptide 10/300 GL column (GE Healthcare, Pittsburgh, PA, USA) at a flow rate of 0.3 mL/min using 0.2 M ammonium hydrogen carbonate as the running buffer. Elution was monitored at 210 nm using a UV detector. LabSolutions 6.108 (Shanghai, China) software was performed for online monitoring and data analysis.

### 4.8. Kinetic Analysis of the Alginate Lyases

The kinetic parameters of AlyC8-CD1 and AlyC8-CD2 for alginate depolymerization were determined by measuring the enzyme activities in the presence of various concentrations (0.3–6 mg/mL) of sodium alginate under their optimal reaction conditions. The A_235_ was recorded to quantify the concentrations of the oligoalginate using a molar extinction coefficient of ϵ = 6150 M^−1^ cm^−1^ [53]. The kinetic parameters *K*_m_ and *V*_max_ were calculated by fitting the data to the Michaelis–Menten equation by non-linear regression via Origin 8.5 software (Northampton, MA, USA). The catalytic constant (*k*_cat_) was calculated by the ratio of *V*_max_ versus enzyme concentration ([E]) [19].

## Figures and Tables

**Table 1:** Kinetic parameters of AlyC8-CD1 and AlyC8-CD2 towards sodium alginate.

Enzyme	*K*_m_ (mg/mL)	*V*_max_ (U/mg)	*k*_cat_ (s^−1^)	*k*_cat_/*K*_m_ (s^−1^ mg^−1^ mL)
AlyC8-CD1	1.64 ± 0.24	24415.9 ± 1974.3	405.66 ± 32.81	247.35
AlyC8-CD2	3.43 ± 0.52	2270.6 ± 160.2	7.81 ± 0.55	2.28

**Table 2:** Primers used in this study.

Gene Product	Primer	Sequence (5′ to 3′) ^a^
AlyC8	AlyC8-F	AAGAAGGAGATATACATATGTGCGGTGGAAGCAGTTCAAA
	AlyC8-R	TGGTGGTGGTGGTGCTCGAGGTTATGTTCAACCCTTAACG
AlyC8-CD1	AlyC8-CD1-F	AAGAAGGAGATATACATATGTGCGGTGGAAGCAGTTCAAA
	AlyC8-CD1-R	TGGTGGTGGTGGTGCTCGAGGATACACCACAACCCAAATC
AlyC8-CD2	AlyC8-CD2-F	AAGAAGGAGATATACATATGATCAGTGGTTCAAACGATTG
	AlyC8-CD2-R	TGGTGGTGGTGGTGCTCGAGGTTATGTTCAACCCTTAACG

^a^ Sequences identical to that of vector pET22b are underlined.

## Data Availability

The genome data of strain C42 has been submitted to the NCBIdatabase under the accession number JAEKGD000000000.1. It can be found here: https://www.ncbi.nlm.nih.gov/nuccore/JAEKGD000000000.1/. The amino acid sequence of AlyC8 has been submitted to the NCBI GenBank database under the accession number MBY7662542.1. It can be found here: https://www.ncbi.nlm.nih.gov/protein/MBY7662542.1.
